# Diagnostic performance of contrast-enhanced ultrasound for acute pyelonephritis in children

**DOI:** 10.1038/s41598-020-67713-z

**Published:** 2020-07-01

**Authors:** Hyun Joo Jung, Moon Hyung Choi, Ki Soo Pai, Hyun Gi Kim

**Affiliations:** 1 Department of Pediatrics, Ajou University School of Medicine, Ajou University Medical Center, 164 World cup-ro, Yeongtong-gu, Suwon, 16499 Republic of Korea; 2 Department of Radiology, Eunpyeong St. Mary’s Hospital, College of Medicine, The Catholic University of Korea, Seoul, 03312 Republic of Korea; 3 Department of Radiology, Ajou University School of Medicine, Ajou University Medical Center, 164 World cup-ro, Yeongtong-gu, Suwon, 16499 Republic of Korea

**Keywords:** Urology, Paediatric research, Kidney, Urinary tract, Paediatric kidney disease, Nephritis

## Abstract

The objective of our study was to evaluate the performance of renal contrast-enhanced ultrasound (CEUS) against the 99m-labeled dimercaptosuccinic acid (DMSA) scan and computed tomography (CT) in children for the diagnosis of acute pyelonephritis. We included children who underwent both renal CEUS and the DMSA scan or CT. A total of 33 children (21 males and 12 females, mean age 26 ± 36 months) were included. Using the DMSA scan as the reference standard, the sensitivity, specificity, positive predictive value, and negative predictive value of CEUS was 86.8%, 71.4%, 80.5%, and 80.0%, respectively. When CT was used as the reference standard, the sensitivity, specificity, positive predictive value, and negative predictive value of CEUS was 87.5%, 80.0%, 87.5%, and 80.0%, respectively. The diagnostic accuracy of CEUS for the diagnosis of acute pyelonephritis was 80.3% and 84.6% compared to the DMSA scan and CT, respectively. Inter-observer (kappa = 0.54) and intra-observer agreement (kappa = 0.59) for renal CEUS was moderate. In conclusion, CEUS had good diagnostic accuracy for diagnosing acute pyelonephritis with moderate inter- and intra-observer agreement. As CEUS does not require radiation or sedation, it could play an important role in the future when diagnosing acute pyelonephritis in children.

## Introduction

Urinary tract infection (UTI) is a common cause of illness with fever in children. It frequently develops in boys during their first year of life and is more frequent in girls of older ages^1^.

Unrecognized and untreated acute pyelonephritis can lead to renal scarring, renal hypertension, and even renal failure^2^. In girls, complications of renal scarring include a higher risk of preeclampsia^3^. Up to 37% of children are reported to have renal scarring after acute pyelonephritis two years after the infection^4^. Therefore, prompt diagnosis and proper management of acute pyelonephritis is crucial.

The current reference standard for renal cortical scar detection after acute pyelonephritis is technetium 99m-labeled dimercaptosuccinic acid (DMSA) renal scintigraphy^5–9^. Computed tomography (CT) can also detect acute pyelonephritis and is a faster imaging tool compared to DMSA scanning^10^. However, these modalities use ionizing radiation and require sedation. Magnetic resonance imaging (MRI) has been introduced as an alternative method to detect acute pyelonephritis in children^11^. Although MRI can be used to detect acute pyelonephritis and is a radiation-free imaging modality, it has limited benefits for young children who need to be sedated to undertake the examination.

Unlike the DMSA scan, CT, or MRI, renal contrast-enhanced ultrasound (CEUS) uses neither ionizing radiation nor sedation and might provide a safer alternative to DMSA scans. In 2014, the European Society of Paediatric Radiology Uroradiology Task Force announced a procedural protocol for renal intravenous CEUS in children^12^. In their recommendations, the indications for renal CEUS included equivocal infection, trauma, vascular and perfusion abnormality, and renal transplants. In 2017, the European Federation of Societies for Ultrasound in Medicine and Biology published guidelines for the non-hepatic application of CEUS^13^. Although the non-hepatic usage of CEUS can still be debated on, a strong consensus has been reached for the use of CEUS to diagnose renal abscess complicated by acute pyelonephritis^13^.

Although renal CEUS, can be a promising alternative to DMSA scans, there have been few studies regarding its clinical application in children. Therefore, this study was carried out to investigate the diagnostic performance of renal CEUS for diagnosing acute pyelonephritis in children. For the investigation, the DMSA scan and CT were used as reference standards.

## Results

### Patients

A total of 33 children were included in this study. There were 21 boys and 12 girls. The mean age of the patients was 26 ± 36 months (range 2–159 months). The mean interval between CEUS and the DMSA scan was 3 ± 3 days. Among the 33 children, there were 13 children who underwent CT. The mean interval between CEUS and CT was 4 ± 2 days. Four patients had negative urinalysis WBC results, but all of the four patients showed positive DMSA results. There were 25 children with positive urine cultures: *Escherichia coli* (n  = 18), coagulase-negative *Staphylococcus* (n = 2), *Enterococcus faecium* (n = 2), *Klebsiella pneumoniae* (n = 1), *Morganella morganii* (n = 1), and *Candida albicans* (n = 1).

No adverse reactions were observed in our study during the 20-min monitoring period after CEUS. There were 27 and 11 children who were sedated for the DMSA scan and CT, respectively. No child was sedated or anesthetized for CEUS.

### Diagnostic accuracy

Diagnostic performances of CEUS are summarized in Table 1. Among the 66 included kidneys, 38 (57.6%) were positive and 28 (42.4%) were negative on the DMSA scan. On CEUS, 42 (63.6%) were positive and 24 (36.4%) were negative. Among the 26 kidneys that underwent CT, 16 (61.5%) were positive and 10 (38.5%) were negative. Per patient basis, 29 among 33, 32 among 33, and 11 among 13 patients were interpreted as positive using the DMSA scan, CEUS, and CT, respectively.

**Table 1:** Diagnostic performance of CEUS for detecting acute pyelonephritis in 66 kidneys.

Reference standard	DMSA scan	CT
Sensitivity	86.8% (33/38)	87.5% (14/16)
Specificity	71.4% (20/28)	80.0% (8/10)
PPV	80.5% (33/41)	87.5% (14/16)
NPV	80.0% (20/25)	80.0% (8/10)
Diagnostic accuracy	80.3%	84.6%

*DMSA* technetium 99m-labeled dimercaptosuccinic acid, *CT* computed tomography, *PPV* positive predictive value, *NPV* negative predictive value.

**Figure 1:**
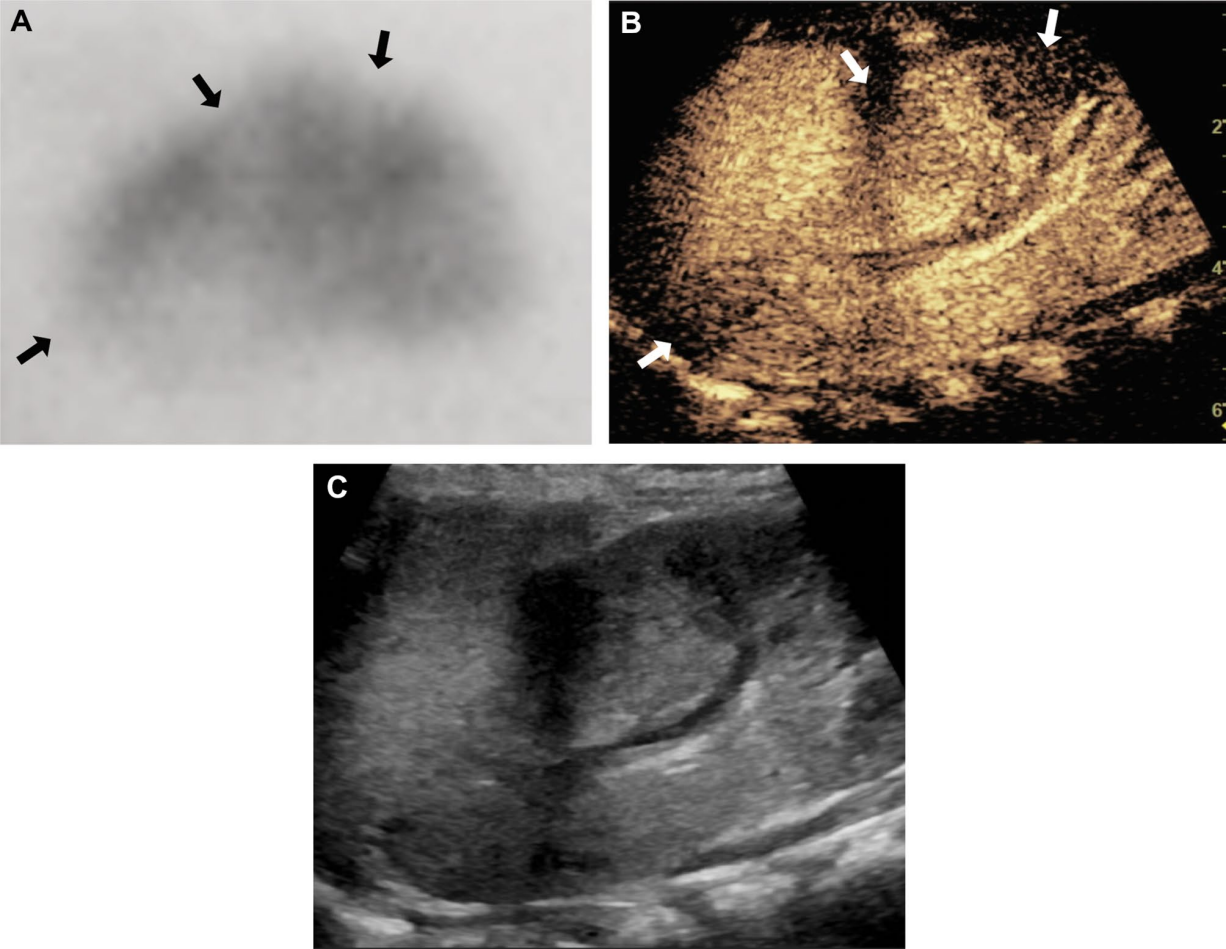
A 9-month-old female with acute pyelonephritis. The DMSA scan image (**A**) shows multifocal cortical defects (arrows) in the left kidney (image rotated so that it corresponds to the CEUS image). The CEUS image on the longitudinal plane (**B**) shows correlating areas of cortical perfusion defects (arrows). The gray-scale image of CEUS (**C**) shows decreased echogenicity in the areas with perfusion defects.

When the DMSA scan was used as the reference standard, sensitivity, positive predictive value (PPV), and negative predictive value (NPV) were fairly high at 80.0% or higher. Specificity was lower at 71.4%. When CT was used as the reference standard, sensitivity, specificity, PPV, and NPV were 80.0% or higher. The sensitivity of CEUS was higher (86.8–87.5%) than the specificity of CEUS (71.4–80.0%). Diagnostic accuracy of CEUS with either the DMSA scan or CT as the reference standard was 80.3% and 84.6%, respectively. A representative true-positive case is illustrated in Fig. 1.

**Figure 2:**
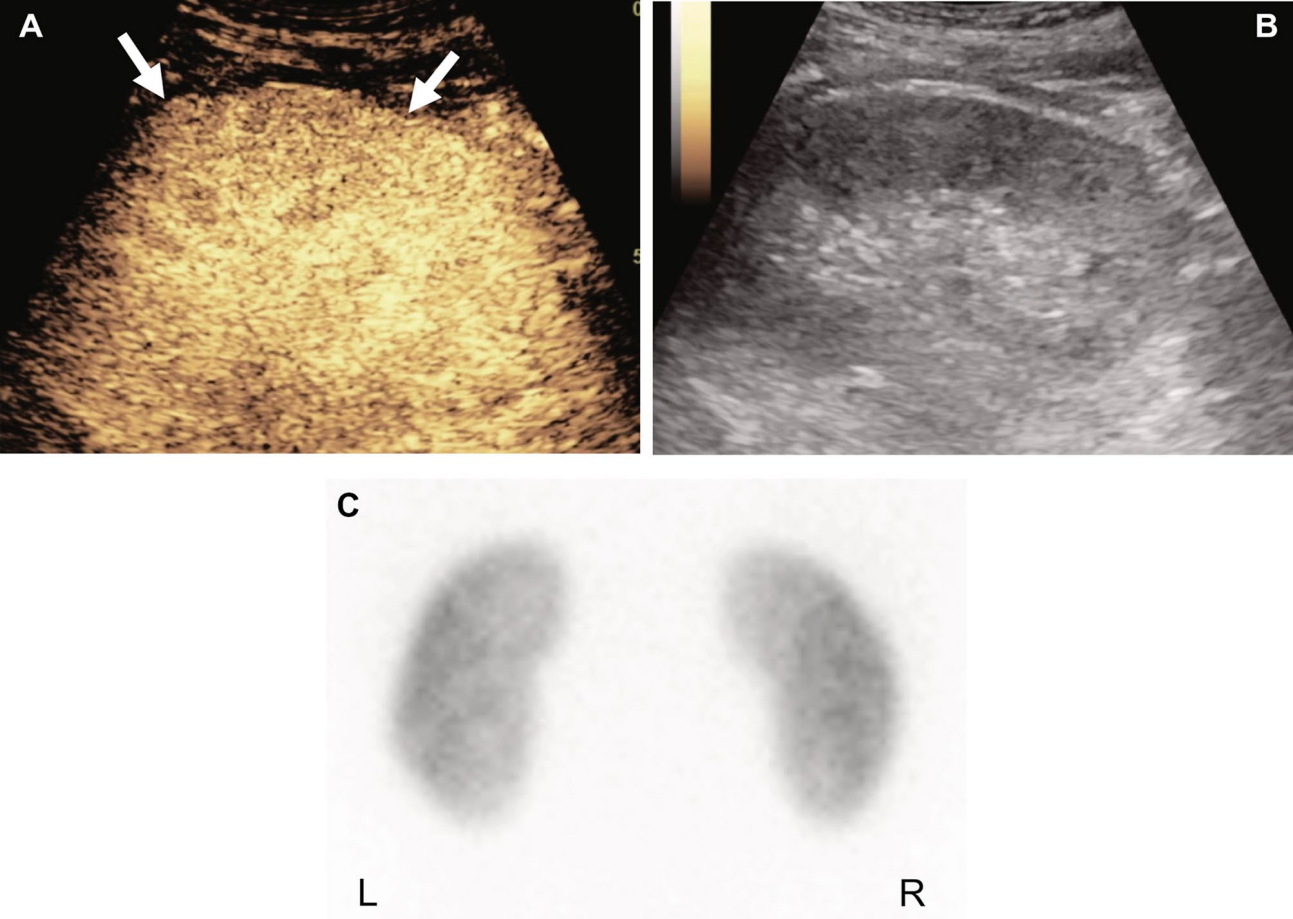
A 13-year-old male admitted for a urinary tract infection. Two radiologists interpreted the CEUS scan of the left kidney with a mid-portion cortical perfusion defect (longitudinal plane, arrow) (**A**, **B**). The posterior view of the DMSA scan was negative (**C**).

**Figure 3:**
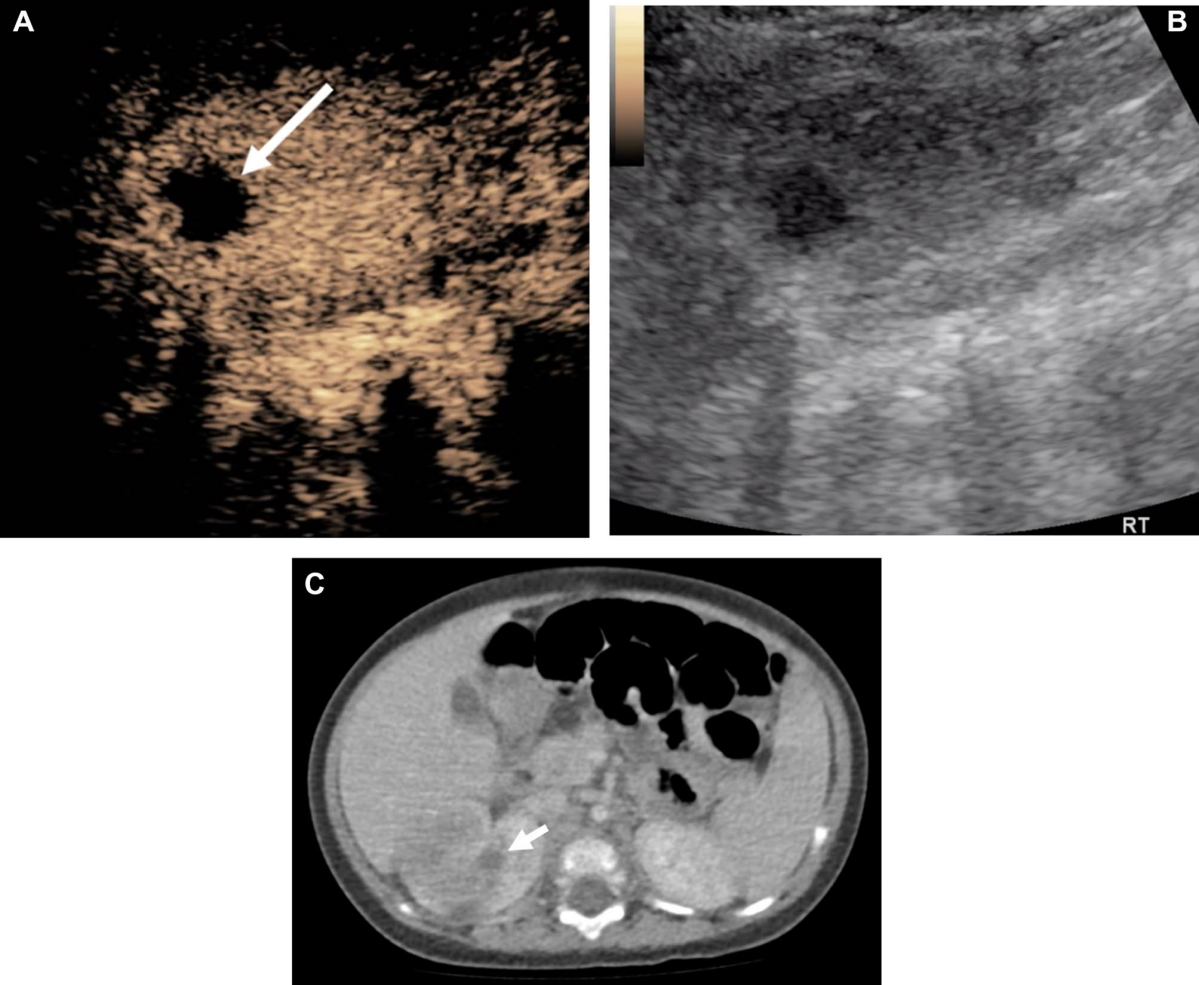
A 5-month-old male with acute pyelonephritis. The CEUS scan on the axial plane (**A**) with the gray-scale image (**B**) shows a hypoechoic lesion at the right kidney cortex (arrow) within the larger area of the hypoenhancing renal parenchyma. The lesion was not enhanced during the enhancement period and interpreted as an abscess. The CT image (**C**) shows a round-shaped non-enhancing lesion (correlating with the abscess on CEUS) (arrow) within a larger area of hypoenhancement (acute pyelonephritis).

Using the DMSA scan as the reference standard, there were 8 false-positive kidneys which showed positive results on CEUS and negative results on the DMSA scan. Among the 8 kidneys, 7 were assessed as positive on CEUS by both radiologists. A representative false-positive case is shown in Fig. 2. There was one kidney with suspected renal abscess on CEUS. The renal abscess was also observed on CT (Fig. 3).

### Inter- and intra-observer variability

Two radiologists evaluated the CEUS of 66 kidneys in 33 children. Both inter-observer and intra-observer agreements for renal CEUS showed moderate agreement with kappa values of 0.54 (p < 0.001) and 0.59 (p < 0.001), respectively.

## Discussion

Previous studies have reported the DMSA scan as the modality of choice for the detection of renal parenchymal lesions^14–16^. In an animal model, the DMSA scan has shown high sensitivity (92%) for diagnosing acute pyelonephritis^17^. However, detection rates vary for the acute stage of the first febrile acute pyelonephritis^18–21^. In addition, using the DMSA scan to detect acute pyelonephritis in infants under 3 months of age is generally discouraged as these infants have immature renal cortices and are open to higher radiation risks^16,19^.

Our institution is a referral hospital and treats approximately a thousand pediatric UTI patients a year. In most cases, acute pyelonephritis can be confidently diagnosed with clinical findings and laboratory tests. Thus, imaging studies should not be the reason for delaying its diagnosis and putting off antibiotic treatment. However, if a patient suffers from recurrent UTI or is suspected to have renal scarring, imaging studies have to be considered. In pediatric UTI patients, imaging studies are particularly important as they may reveal underlying conditions such as vesicoureteral reflux or congenital anomaly. In some children, an accurate diagnosis of acute pyelonephritis will not be possible with clinical signs and laboratory test results, leading to the need for imaging diagnostic tools such as DMSA scans^16^. All of the patients with negative urinalysis results in our study showed positive results on DMSA scans. Although it is the modality of choice for detecting renal parenchymal lesions, the primary disadvantage of DMSA scans is the inevitable exposure of ionizing radiation to children, which is about 2.84 mSv^22^. Additionally, due to shortages in the supply of the DMSA radiotracer, the availability of the DMSA scan itself has at times been inconsistent^6^. Therefore, there is a need to develop alternative imaging tools for diagnosing acute pyelonephritis.

In our study, the sensitivity for detecting acute pyelonephritis using CEUS was as high as 86.8–87.5%. The sensitivity of our results is similar or higher than the sensitivities of previous reports using power Doppler ultrasound (US) to evaluate acute pyelonephritis, which ranged 23–89%^7,18–20,23^. Power Doppler US has been considered an imaging technique for the diagnosis of acute pyelonephritis, but it may miss renal parenchymal changes in acute pyelonephritis due to limited detection of low flow^24^. CEUS, on the other hand, has a higher sensitivity for the detection of renal parenchymal lesions^23^. This is because an acute pyelonephritis diagnosis requires a technique that is able to study the microcirculation of the kidney parenchyma, which is at a sensitivity level that cannot be reached by power Doppler US^25^.

Previous studies with adults showed a higher sensitivity (90–95%) for diagnosing acute pyelonephritis using CEUS compared to our study^23,26^. One of these past studies evaluated acute pyelonephritis in transplanted kidneys and used enhanced MRI as the reference standard^26^. Another compared the sensitivities of conventional US, power Doppler US, and CEUS for the detection of acute pyelonephritis and used correlation of imaging findings, clinical data, and laboratory findings as the reference standard^23^. The authors of this past study showed higher sensitivity with CEUS (90%), compared to conventional US (57%) and power Doppler US (23%)^23^. Factors that could have decreased the sensitivity of CEUS in our study include infant crying, breathing motions, intestinal gas, or rib artifacts. If we consider these factors, we can expect motion-free adults with or without transplanted kidneys in the anterior location to show a higher sensitivity for acute pyelonephritis than our results.

The specificity of CEUS for detecting acute pyelonephritis lesions with the DMSA scan as the reference standard was lower than the sensitivity of CEUS. Previous studies using power Doppler US to evaluate acute pyelonephritis showed a specificity of 53–95%^7,18–20^. According to another study on adults, the specificity for detecting acute pyelonephritis (80%) did not differ between conventional US, power Doppler US, and CEUS^23^. Adding axial plane images would have helped differentiate hypoperfusion areas more accurately. On the other hand, we speculate that the specificity of CEUS was underestimated in our study. In our study, 7 among 8 kidneys that were false-positive were assessed as acute pyelonephritis by both radiologists. This could be due to the higher sensitivity of CEUS for diagnosing acute pyelonephritis. Although we choose the DMSA scan as the reference standard, its sensitivity varies in children, especially in young children^27^. If these false-positive cases had actually been true-positive cases, regardless of the DMSA results, the specificity of CEUS would have been higher. This assumption is supported by the higher specificity of CEUS using CT as the reference standard (80.0% vs 71.4%) in our study. However, to validate this assumption, further studies with pathologic confirmation are needed.

Using CEUS, we found one kidney with renal abscess. There was a previous study showing the role of CEUS for diagnosing renal abscess^28^. As in our study, the abscesses were not enhanced throughout the enhancement period on CEUS^28^. The detection of abscesses is a critical role of imaging when evaluating patients with acute pyelonephritis. CT is more sensitive than US for the detection of renal abscess, but children are exposed to significant radiation doses during the CT scan^10^. MRI can be another imaging modality for diagnosing acute pyelonephritis and renal complications in children without ionizing radiation^11,29^. Sophisticated management of MRI scanning can reduce the usage of sedation as well^29^. Still, the role of MRI in pediatric acute pyelonephritis subjects needs further validation and we think CEUS could potentially substitute the DMSA scan in the future.

Inter- and intra-observer variability showed moderate agreement (kappa = 0.54 and 0.59, respectively) for CEUS diagnosing acute pyelonephritis. Although moderate agreement might not seem to be enough to support CEUS as an accurate diagnostic tool, our inter-observer agreement was similar to that of a previous study using the DMSA scan (kappa = 0.54–0.63) or SPECT (kappa = 0.54–0.59) for renal cortical lesions in children^8^. A previous study using MRI with diffusion-weighted imaging to detect acute pyelonephritis in children showed higher inter-observer agreement (kappa = 0.79)^30^. Inter-observer variability is an important scale of reliability for imaging modalities. In our study, the recorded CEUS movie images were retrospectively interpreted by two radiologists. If CEUS was performed and interpreted in real time, a higher diagnostic confidence might have been achieved. This is because like other US techniques, CEUS is performer dependent, and affected by the management and skills of the performer. Higher diagnostic confidence could have led to higher agreement. On the other hand, lack of skills in the US performer might lead to decreased confidence and potentially decrease agreement. However, as only one pediatric radiologist performed CEUS in this study, we could not evaluate variability in terms of US performance. In addition, the DMSA reports used as the reference standard did not include the specific location of the cortical defect, and the location of the defect was not evaluated on CEUS. The reproducibility of using CEUS to diagnose acute pyelonephritis in children should be further validated with a larger number of cases and observations.

There are several limitations in our study. First, there were technical limitations which could cause inhomogeneous enhancement during CEUS. Although we set the mechanical index as low as possible in every study, values may vary depending on the depth of the kidney portion in question. Besides, different levels of abdominal compression and residual microbubbles in the second-examined kidney will also result in different enhancement patterns. Second, we used the DMSA scan as the reference standard, but its diagnostic accuracy might not be regarded as high enough to be considered a reference standard. We speculate that the specificity of CEUS was underestimated in our study. As mentioned earlier, pathologic confirmation and comparison of the diagnostic performances of different modalities should be evaluated in future studies. Third, there were no reports on the locations of defects on DMSA scans and CEUS studies and the two studies were not conducted on the same day. As the locations of the defects could not be analyzed, we cannot be sure whether the two studies were displaying the defects of the same lesion in the same location. The mean interval between the two studies was as short as 3 days, but patients were already being treated with antibiotics during the admission process. Therefore, their inflammatory conditions could have changed between the DMSA scan and CEUS. Last, the DMSA scan-positive rate was relatively high (57.6%). DMSA scan-positive rates from previous studies were 28–79%^18–21^. Per patient basis, there were 29 among 33 patients who showed positive DMSA scan results. Higher positive rates could have resulted in higher sensitivity. Our institution is a tertiary hospital and clinicians selected patients for CEUS when the patients were suspected of atypical recurrent UTI or renal complications due to acute pyelonephritis. The selection process itself could have resulted in a skewed patient population. There is potential for some patients to have both renal scarring and acute pyelonephritis on DMSA scans. The additional benefit of CEUS over DMSA scanning would be higher resolution that enables differentiation between renal scarring and acute pyelonephritis. Therefore, the benefits of CEUS for detecting residuals, scars, or perfusion defects in children who do not respond well to treatment or who have recurrent UTI should be further evaluated in future studies.

In conclusion, our study showed that renal CEUS has good sensitivity, specificity, and diagnostic accuracy for diagnosing acute pyelonephritis in children. Inter-observer and intra-observer agreements were moderate. As renal CEUS does not require ionizing radiation and patient sedation for scanning, it might play an important role in the future when diagnosing acute pyelonephritis in children.

## Methods

This retrospective study was approved by Institutional Review Board of Ajou University Hospital and the requirement for patient consent was waived. Informed consent for the off-label usage of the US contrast agent was obtained from all patient guardians. The study was conducted according to the guidelines of the Declaration of Helsinki.

### Subjects

We retrospectively reviewed pediatric patients (15 years old or younger) who were admitted to our institution for suspected acute pyelonephritis from August 2017 to August 2018 and who underwent both CEUS and DMSA scanning during their hospital stay. Some of these patients underwent CT in the emergency room for prompt diagnosis and management before admission. Patients were diagnosed with UTI if they had fever (≥ 37.8 °C), pyuria, and positive urine culture^9^. Some patients who were pretreated with antibiotics did not fulfill the abovementioned criteria at admission but showed a positive urine culture on a follow-up exam. The need for CEUS was determined by the clinician if the patient suffered from recurrent UTI and/or was suspected to have complications due to acute pyelonephritis. Patients who had any of the following were excluded from the CEUS study: (1) previous history of an allergic reaction to the US contrast agent, (2) left-to-right shunt with congenital heart disease, (3) respiratory distress syndrome, (4) severe pulmonary hypertension, and (5) severe systemic hypertension. Administration of sedation or anesthesia for the imaging studies were recorded.

### CEUS

All renal CEUS studies were performed by one pediatric radiologist. The radiologist reviewed the history and previous imaging studies of each patient before conducting CEUS. First, the patients underwent conventional non-enhanced renal ultrasound using a LOGIQ E9 or LOGIQ E10 (GE Healthcare, Milwaukee, WI, USA) ultrasound machine. Either a curved 1–6 transducer or curved 3–10 transducer was used depending on body size. Second, the patients underwent renal CEUS using either a linear 2–9 or curved 1–6 transducer. The mechanical index was set as low as possible from 0.09 to 0.12^31^. All subjects were administered the SonoVue (Bracco, Milan, Italy) contrast agent through a peripheral IV line. Patients younger than 3 years of age received 0.07—0.1 ml/kg body weight of contrast agent and those older than 3 years received 0.06 ml/kg body weight of contrast agent for each injection according to a previous guideline for pediatric urinary tract CEUS studies^12^. Each injection was immediately followed by a 5-ml normal saline solution flush. The time window was 0–3 min for each kidney following the previous guideline^12^. During the time window, the performer scrolled the transducer to completely cover both kidneys. The longitudinal plane was chosen as the standard view, but the axial plane was also chosen whenever the performer found it necessary to further visualize lesions in the kidneys. Patients were in the supine or oblique lateral position during the study. One injection was done for each kidney. Two different injections were given with a 5-min interval. Between the two exams, we used “flash-replenishment” to remove residual microbubbles from the second kidney. After renal CEUS, all patients were observed for 20 min for any adverse reactions to the contrast agent.

### DMSA scan

DMSA scans were performed with a gamma camera (Siemens Orbiter, Erlangen, Germany) after the intravenous injection of an adult-equivalent dose of 37-MBq technetium-99 DMSA with weight-adjustment. Single-photon emission computed tomography was performed 4 h after the isotope was administered for a scan time of 20 min.

### CT

Single-phase enhanced abdominal CT was performed with a 128 MDCT scanner (SOMATOM Definition Edge, Siemens Healthcare, Erlangen, Germany). The scan parameters were as follows: 100 kVp, 80 mAs with automatic tube current modulation (reference mA), rotation time 0.5, and pitch 1.2. Automatic tube current modulation systems adapt the tube current according to each patient’s attenuation characteristics. The image quality metric is specified in the form of either image noise or reference mAs and we set the reference mAs as 80 mAs^32^. Images were reconstructed at 3 mm slice thickness. Intravenous contrast Bonorex Iohexol 300 (Central Medical Services, Seoul, Korea) was administered at an average dose of 1.5–2 ml/kg body weight. The nephrogenic phase was acquired 60–90 s after the contrast was injected depending on patient age, acquisition time, contrast amount, and injection speed^33^.

### Image analysis

All the reviewers were blinded to prior interpretations and prior imaging studies.

CEUS movie files consisting of one clip for each kidney were generated by a radiologist. The analyzed movie file had a renal CEUS time window of 1–2 min instead of the whole 3 min of the original file as the late parenchymal phase has been reported to be the best phase for pyelonephritis^28^. To evaluate inter-observer variability, the movie clips were randomly displayed and interpreted by two faculty radiologists. Radiologist 1 was a pediatric radiologist and Radiologist 2 was an abdominal radiologist, both with 9 years of experience in radiology and 2 years of experience in CEUS. The radiologists were blinded to other imaging findings and clinical information. Decreased cortical perfusion was considered to indicate acute pyelonephritis. Each radiologist documented the presence of acute pyelonephritis (absent or present) and its location (right or left kidney). Radiologist 1 repeated assessments two weeks later to evaluate intra-observer variability. The presence or absence of abscess was also determined and recorded. A renal abscess was diagnosed when there was a hypoechoic lesion without enhancement during the CEUS study^28^.

CT images were interpreted by Radiologist 1. A typical wedge-shaped cortical area of decreased enhancement was considered to indicate acute pyelonephritis^10^. Again, the presence of acute pyelonephritis and its location (right or left kidney) was documented. The presence or absence of renal abscess was also documented. A renal abscess was diagnosed when there was a well-defined low attenuating mass with wall enhancement^34^.

DMSA scan reports, which were wrote up by one of the nuclear physicians in our institution, were used for this study. The International Radionuclide Nephrourology consensus criteria were used to interpret DMSA scans and the scans were deemed positive if there were any cortical defects^35^.

### Statistics

Reports from DMSA scans and CT served as reference standards for the agreement analysis. Sensitivity, specificity, PPV, and NPV were calculated for CEUS with the DMSA scan as the reference standard and CEUS with CT as the reference standard. The generalized kappa value was used to assess agreements for inter- and intra-observer variability. Agreements for inter- and intra-observer variability were categorized as follows: 0.81–0.99, almost perfect agreement; 0.61–0.80, substantial agreement; 0.41–0.60, moderate agreement; 0.21–0.40, fair agreement; 0.01–0.20, slight agreement, and < 0.01, poor or less than chance agreement^36^. A p value of less than 0.05 was considered statistically significant. SPSS v.25.0 (SPSS, Chicago, IL, USA) was used for analysis.
